# Occurrence and characteristics of suicidal ideation in psychiatrically healthy individuals based on ecological momentary assessment

**DOI:** 10.1038/s41380-024-02560-2

**Published:** 2024-05-10

**Authors:** Maria A. Oquendo, Hanga C. Galfalvy, Tse-Hwei Choo, Sarah Herzog, Ainsley K. Burke, M. Elizabeth Sublette, J. John Mann, Barbara H. Stanley

**Affiliations:** 1 Perelman School of Medicine, University of Pennsylvania, Philadelphia, PA USA; 2 Vagelos College of Physicians and Surgeons, Columbia University and New York State Psychiatric Institute, New York, NY USA

**Keywords:** Depression, Psychology

## Abstract

Decedents with no known mental disorder comprise 5–40% of suicides, suggesting that suicide ideation (SI) and behavior may occur in the psychiatrically healthy with important implications for suicide risk screening. Healthy Volunteers (HV) and patients with Major Depressive Disorder (MDD) provided 7 days of Ecological Momentary Assessment (EMA) data about SI and stressors. Longitudinal mixed effects logistic regression models compared HV and patient SI and stressors. Mixed effects linear regression models compared HVs’ and patients’ SI score change from the previous epoch’s SI score when each stressor occurred. HVs (*n* = 42) reported less frequent (*p* < 0.001) and less intense SI (*p* < 0.003) than patients (*n* = 80), yet did endorse SI and/or SI-related items in 44% of EMA epochs, endorsing SI items in 25% of epochs with non-zero SI scores. For 7 of 8 stressors, patients reported stressors more often than HVs (all *p* < 0.001) responding to them with increased SI (0.0001 < *p* < 0.0472). HVs were relatively resilient to stressors, reporting SI increases only in response to neglect (*p* < 0.0147). Although SI and SAs are documented among psychiatrically healthy individuals, scientific attention to these observations has been scant. Real-time SI measurement showed that HVs’ SI was less pronounced than MDD patients’, but was endorsed, nonetheless. Patients were more likely to report stressors than HVs, perhaps due to greater sensitivity to the environment, and reported SI in response to stressors, which was less common in HVs. Both MDD patients and HVs most often manifested passive SI (viz, “decreased wish to live”). However, passive SI (viz, “desire for death”), may predict suicide, even absent SI per se (thinking about killing yourself). This study validates the utility of real-time SI assessment, showing that HVs endorse SI items in 11% of epochs, which implies that suicide risk screening focused on those with mental disorders may be too narrow an approach.

## Introduction

Suicide is a leading cause of death world-wide, and the World Health Organization (WHO) estimates that about 700,000 persons died by suicide in 2019 [1]. Moreover, in the US, in 2021, for every suicide, there are about 38 nonfatal suicide attempts [2]. The frequency of suicidal ideation (SI) is even greater than that of nonfatal suicide attempts, and SI rates have increased over the last decade. US data from 2009 representative of the general population indicate that 3.7% of the population had suicidal thoughts in the preceding year [3]. By 2019, a population-based study using the same instrument and including more than 250,000 individuals found a more than 16% increase in the number of individuals reporting SI (4.3% of the US population) [4], translating into over 14 million people experiencing SI in the previous 12 months.

In Western countries, suicide decedents with no known psychiatric diagnosis comprise 5–40% of suicides [5] even when the decedent’s condition is assessed using psychological autopsy [6], shown to be reliable for both Axis I and II disorders [7, 8]. It stands to reason then, that there exist individuals who experience suicidal ideation (SI) who do not have a detectable psychiatric diagnosis or do not meet criteria for a psychiatric diagnosis, and that SI in this group can be significant enough to result in suicide.

Many, but not all [9, 10], studies have reported SI in healthy volunteers (HV) documented not to have a psychiatric diagnosis [11–15], even among HVs who were screened to ensure they had no lifetime suicide attempts [16, 17]. However, these are usually small samples of controls recruited as a comparison group for clinical or biological studies of patients with a variety of psychiatric conditions. A limitation is that some studies used only one item in a depression scale to measure SI [13, 18], while others administered a retrospective scale to measure SI at a single time point [16, 17]. Consequently, information about the frequency and severity of SI and the circumstances surrounding SI in HVs is limited and estimates about the occurrence of SI in psychiatrically healthy persons in the general population do not exist, to our knowledge. Of note, a study of UK Biobank volunteers, a sample known to have a bias towards including healthier individuals, revealed that 12% of individuals reporting self-harm (both suicidal and non-suicidal self-injury) did not meet criteria for a psychiatric diagnosis [19]. The observation that at least 5% of suicides appear not to have a psychiatric diagnosis [6] suggests that a similar proportion of suicide ideators also may not have or appear to have a diagnosis. Five percent of the 14 million suicide ideators in the US would translate into about 700,000 individuals with SI but no psychiatric diagnosis, a non-trivial number of individuals with yet unrecognized risk of suicide.

Leveraging data from a study of SI and suicidal behavior that included healthy volunteers (HVs) and individuals with Major Depressive Disorder (MDD), we tested the hypotheses that: (1) Some HVs, carefully screened to exclude individuals meeting lifetime criteria for Axis I or II disorders and those with a personal or family history of suicidal behavior, would endorse SI on ecological momentary assessment (EMA); and (2) HVs’ SI scores in response to stressors would be lower than those of MDD patients. We also explored whether HV and MDD groups differ in terms of SI in response to stressors. If some HVs indeed exhibit SI, then suicide prevention strategies that rely on screening would be more effective if screening were expanded beyond those with known mental health conditions.

## Participants and methods

### Sample

Healthy volunteers (HVs) and patients with Major Depressive Disorder (MDD) were recruited through advertisements or in the emergency department (patients only). All participants provided written informed consent. Power calculations were conducted prior to grant submission.

#### Healthy volunteer sample

HVs (N = 42) were free of any clinical DSM-IV Axis I or II diagnoses [20, 21] except for simple phobia, were aged 18–65 years, and were physically healthy. Further exclusion criteria for HVs were (1) family history of suicidal behavior or Major Depression; (2) lifetime IV-drug use; (3) lifetime MDMA use (>3 times). Nicotine users were not excluded from either group.

#### MDD sample

MDD participants (N = 80) met DSM-IV [21] criteria for a current major depressive episode and were aged 18–65 years. Exclusion criteria were (1) substance/alcohol abuse (past 2 months), or past-year substance/alcohol dependence; (2) greater than 3 lifetime incidents of 3,4-methylenedioxy-methamphetamine (MDMA) use; (3) lifetime intravenous drug use; (4) lifetime psychosis (5) past-year anorexia or bulimia nervosa; (6) first-degree relative with schizophrenia (for those < 33 years old); (7) significant active physical illness; (8) recent (past 6 months) electroconvulsive therapy; (9) head trauma leading to cognitive impairment or loss of consciousness.

#### Clinical assessment

Psychiatric diagnoses, or lack thereof, were established for patients and HVs based on the Structured Clinical Interview for DSM-IV [21], conducted by psychologists with a doctoral- or masters’-level degree who were trained to a criterion level in diagnostic reliability, and confirmed in consensus conference. Depression severity was assessed with the 17-item Hamilton Depression Rating Scale (HDRS-17) [22] and the Beck Depression Inventory [23]. Hopelessness was assessed with the Beck Hopelessness Scale [24]. Reasons for living were assessed using the Reasons for Living Inventory [25]. Childhood Trauma was assessed using the Childhood Trauma Questionnaire (CTQ) [26] and its 5 subscales. Aggression, hostility, and impulsivity were assessed using the Brown-Goodwin Aggression History [27], the Buss-Durkee Hostility Inventory [28], and the Barratt Impulsivity (11) Scale [29], respectively.

#### Ecological momentary assessment

EMA has the advantage of allowing assessment in “real time” minimizing recall bias and collecting data proximal to the occurrence of an event. It also limits the confounds of data collection in the context of a laboratory and data collection using smartphone technology, which is second nature for a large segment of the population, makes it relatively unobtrusive. We trained participants on ecological momentary assessment (EMA) using either their own smartphone or a personal digital device provided by the study team. During a week-long EMA period (7 consecutive days), participants reported on suicidal ideation and stressors six times daily. Each day had a designated 12-h wake period customized to each participant, divided into 2-h epochs (N = 6). Within each 2-h epoch, prompts were presented at random intervals to ensure that data collection was not impacted by a participant’s fixed schedule (e.g., sleep, meals) on a regular basis. SI was assessed with nine items adapted from the Scale for Suicidal Ideation [30]. Participants received a prompt reading “Please indicate if any of the following events occurred since the last prompt,” and rated the intensity with which they experienced each SI item on a Likert scale that spanned from 0 (“very slightly or not at all)” to 4 (“extremely”). The items included: “thoughts about dying;” “a wish to live;” “a wish to die;” “a wish to sleep and not wake up;” “suicidal thoughts as a wish to escape;” “like there were reasons for living;” “thoughts about hurting yourself;” “an urge to hurt yourself;” and “thoughts about killing yourself (suicidal thoughts).” We considered 7 of the items actual SI (“thoughts about dying,” “a wish to die;” “a wish to sleep and not wake up;” “suicidal thoughts as a wish to escape;” “thoughts about hurting yourself;” “an urge to hurt yourself;” and “thoughts of killing yourself”) and the other two as SI-related (“a wish to live [reverse coded];” “like there were reasons for living [reverse coded]”). Participants also were queried about the occurrence (yes/no) of the following stressors since the last prompt: (1) disagreement; (2) rejection; (3) interpersonal disappointment 4) compliment; (5) neglect; (6) loss; (7) bad news; and (8) painful reminder from the past.

For HVs, the average number of EMA epochs with responses was 31.9 (Range 10–41); for patients it was 30.3 (range 10–44) (participants with fewer than 10 EMA observations were excluded). Delayed responses to prompts and unprompted responses that were at least 20 min after the previous response (the participant entered responses despite not receiving a prompt from the smart device) were included. Total SI scores, computed by adding the 9 SI item scores within a given epoch, yielded a time-varying total SI score. Change in total SI at a given time *t* was computed as the difference between the total SI score at time *t* and the total SI score at the previous epoch, (*t*-1), provided both observations occurred on the same day. A time-varying stressor indicator was also computed to identify epochs with and without stressors (Yes/No). SI change following epochs with stressors was regarded as stress-reactive SI.

The New York State Psychiatric Institute’s Institutional Review Board approved all study procedures and informed consent forms.

#### Statistical analysis

Patient and HV demographic and clinical variables were compared using Chi-square tests or t-tests as appropriate. Wilcoxon rank test for clustered data compared the distribution of EMA SI scores in HVs and patients over time using the “clusrank” package in R [31]. Longitudinal mixed effects logistic regression models, with an AR(1) correlation structure for within subject observations, compared HVs’ and patients’ reports of stressors and of SI, with the log odds of a reported stressor or of any reported SI modeled as the outcome, and an indicator for patient vs HV as the predictor. Separate models were fit for each of the stressors and for SI. Mixed effects linear regression models, also with AR(1) covariance structure as well as random intercepts for subject, with variances allowed to differ between HVs and patients, compared HVs’ and patients’ SI score change from the previous epoch’s SI score when each of the 8 stressors occurred relative to no event. Change in SI from the previous epoch was modeled as the outcome, with the patient/HV indicator, reported stressor, and the interaction of the patient/HV indicator and the stressor as the predictors. Again, separate models were fit for each stressor. All analyses were performed using SAS version 9.4 unless otherwise noted.

## Results

### Participant characteristics

HV and patient groups had a comparable preponderance of women (59% and 64%, respectively), and groups did not differ in terms of sex, race or ethnicity. As expected, they differed in clinical characteristics (see Table 1). Patients had higher scores on the 17 item Hamilton Depression Scale, the Beck Depression Inventory and the Beck Hopelessness Inventory, relative to HVs. They also reported between 25% and 71% more childhood trauma on CTQ subscales and 39% more lifetime aggression, 79% more hostility and 54% more impulsiveness. HVs reported more reasons for living.

**Table 1:** Demographic and clinical characteristics of healthy volunteers and patients.

	Total sample		Healthy volunteers		Patients		
	(*n* = 122)		(*n* = 42)		(*n* = 80)		
Variables	*n*	%	*n*	%	*n*	%	*p*
Sex	.		.		.		0.452
Male	44	36.40%	17	41.50%	27	33.80%	.
Female	75	62.00%	24	58.50%	51	63.80%	.
Trans: Female to Male	2	1.70%	0	0.00%	2	2.50%	.
Race	.		.		.		0.051
Asian	16	13.30%	6	14.60%	10	12.70%	.
Black or African American	37	30.80%	17	41.50%	20	25.30%	.
White	57	47.50%	15	36.60%	42	53.20%	.
More than one race	8	6.70%	1	2.40%	7	8.90%	.
Unknown or not reported	2	1.70%	2	4.90%	0	0.00%	.
Ethnicity	.		.		.		0.71
Hispanic	29	24.00%	9	22.00%	20	25.00%	.
Not Hispanic	92	76.00%	32	78.00%	60	75.00%	.
Actual attempt	.		.		.		**<0.0001**
At least 1 actual attempt	29	24.00%	0	0.00%	29	36.30%	.
No actual attempts	91	75.20%	40	100.00%	51	63.80%	.
Hamilton Depression Scale 17 Item [Mean (SD)]	122	11.0 (8.7)	42	1.4 (1.7)	80	16.0 (6.2)	**<0.0001**
Beck Depression Inventory [Mean (SD)]	119	15.1 (13.0)	41	1.6 (3.8)	78	22.2 (10.1)	**<0.0001**
Emotional Abuse [Mean (SD)]	115	10.6 (5.1)	39	7.3 (3.0)	76	12.3 (5.2)	**<0.0001**
Physical Abuse [Mean (SD)]	115	8.2 (4.8)	39	6.6 (2.8)	76	9.0 (5.4)	**0.003**
Sexual Abuse [Mean (SD)]	115	7.8 (5.3)	39	6.4 (4.5)	76	8.5 (5.5)	**0.041**
Emotional Neglect [Mean (SD)]	115	11.6 (5.3)	39	8.4 (3.5)	76	13.3 (5.3)	**<0.0001**
Physical Neglect [Mean (SD)]	114	7.3 (2.9)	39	6.3 (2.3)	75	7.9 (3.0)	**0.004**
Barratt Impulsivity Scale [Mean (SD)]	115	44.6 (18.8)	39	32.9 (15.0)	76	50.6 (17.7)	**<0.0001**
Brown-Goodwin Aggression Scale [Mean (SD)]	117	15.0 (5.0)	40	11.9 (2.6)	77	16.6 (5.1)	**<0.0001**
Beck Hopelessness Inventory [Mean (SD)]	119	7.6 (6.7)	41	1.3 (1.6)	78	10.9 (5.9)	**<0.0001**
Buss Durkee Hostility Inventory [Mean (SD)]	114	28.4 (13.2)	38	18.6 (11.5)	76	33.3 (11.2)	**<0.0001**
Reasons for Living Inventory [Mean (SD)]	111	173.7 (45.8)	39	202.7 (30.1)	72	158.0 (45.3)	**<0.0001**

Bold font indicates that the difference is statistically significant.

### Suicidal ideation scores in healthy volunteers and patients

Among HVs, the range of total SI scores was 0–23. Most EMA epochs (56%) evinced no SI (768/1378); 44% (610) had at least one positive score. The median EMA total SI score for the 1378 epochs was 0 (“very slightly or not at all”) with an interquartile range (IQR) of 0 to 4. The median EMA total score for each one of 9 SI items measured once for the whole week of EMA, and rated at the end of the EMA, was also 0.

MDD patients had higher total SI scores compared with HVs, ranging from 0 to 36, (*Z* = 2.97, *p* = 0.0030) with a median of 5 (IQR: 2–8) based on data from 2296 epochs. Compared to HVs, a much smaller proportion (325/2296, 14%) of epochs yielded no SI. The median score was 0 for 6 of 9 SI items. The exceptions were “a wish to live” and “like there are reasons for living” [both reverse coded], which had medians of 2 and interquartile ranges (IQR) of 0–3 and 1–3, respectively, and “suicidal thoughts as a wish to escape” which had a median of 1 and an IQR of 0–3. Longitudinal mixed effects logistic regression models indicated that patients with MDD reported SI more frequently than HVs (OR = 7.7, *p* < 0.0001) and more frequently endorsed each of the individual SI item than HVs with ORs ranging from 4.9 to 35.3 (*p* < 0.0001 for all items) (see Table 2).

**Table 2:** Proportion of responses indicating SI during EMA epochs and odds ratio that patients would report SI compared to HVs.

	Patients		HVs		Difference		
SI Item (Any vs None)	Proportion		Proportion		Odds ratio		
Label	Est	95% CI	Est	95% CI	Est	95% CI	*P* > |t|
Thoughts about dying	18.80%	16.13–21.80%	0.65%	0.34–1.25%	35.28	17.78–70.00	<0.0001
A wish to live	73.38%	68.28–77.93%	32.83%	27.18–39.01%	5.64	3.91–8.13	<0.0001
A wish to die	20.20%	17.13–23.66%	1.02%	0.58–1.78%	24.66	13.51–45.02	<0.0001
A wish to sleep and not wake up	32.64%	28.7–36.83%	1.45%	0.94–2.23%	32.92	20.42–53.06	<0.0001
Suicidal ideation as wish to escape	61.59%	57.41–65.61%	5.15%	3.58–7.36%	29.51	19.43–44.81	<0.0001
Like there were reasons for living	76.52%	71.82–80.64%	39.90%	34.12–45.97%	4.91	3.46–6.96	<0.0001
Thoughts about hurting yourself	12.31%	10.20–14.79%	1.09%	0.66–1.8%	12.76	7.35–22.16	<0.0001
An urge to hurt yourself	10.14%	8.24–12.43%	1.10%	0.62–1.95%	10.14	5.42–18.98	<0.0001
Thoughts about killing yourself	11.06%	9.00–13.53%	0.58%	0.29–1.16%	21.33	10.22–44.51	<0.0001
Ideation Total	86.15%	82.70–89.00%	44.62%	39.00–50.39%	7.72	5.44–10.96	<0.0001
**Proportion of responses indicating occurrence of stressors during EMA epochs and odds ratio that patients would report an event relative to HVs**.							
	**Patients**		**HVs**		**Difference**		
Stressors (yes/no)	**Proportion**		**Proportion**		**Odds Ratio**		
Label	**Est**	**95% CI**	**Est**	**95% CI**	**Est**	**95% CI**	**P** > **|t**|
Disagreement	10.24%	8.91–11.75%	3.21%	2.29–4.48%	3.45	2.36–5.04	<0.0001
Rejection	8.21%	6.95–9.68%	0.51%	0.24–1.07%	17.53	8.13–37.82	<0.0001
Interpersonal disappointment	21.60%	19.45–23.91%	2.61%	1.86–3.65%	10.27	7.09–14.86	<0.0001
Compliment	23.46%	21.21–25.86%	22.20%	19.28–25.42%	1.07	0.86–1.34	0.5221
Neglect	18.33%	16.13–20.76%	1.67%	1.07–2.59%	13.21	8.22–21.22	<0.0001
Loss	7.50%	6.09–9.20%	1.32%	0.74–2.35%	6.04	3.23–11.26	<0.0001
Bad news	11.20%	9.71–12.89%	2.04%	1.36–3.06%	6.05	3.88–9.44	<0.0001
Painful reminder	34.51%	31.58–37.57%	3.56%	2.67–4.73%	14.29	10.33–19.78	<0.0001

*EMA* Ecological Momentary Assessment, *HV* healthy volunteer, *CI* confidence interval, *Est* estimate, *SI* suicidal ideation

### Characteristics of Suicidal Ideation in Healthy Volunteers

**Fig. 1:**
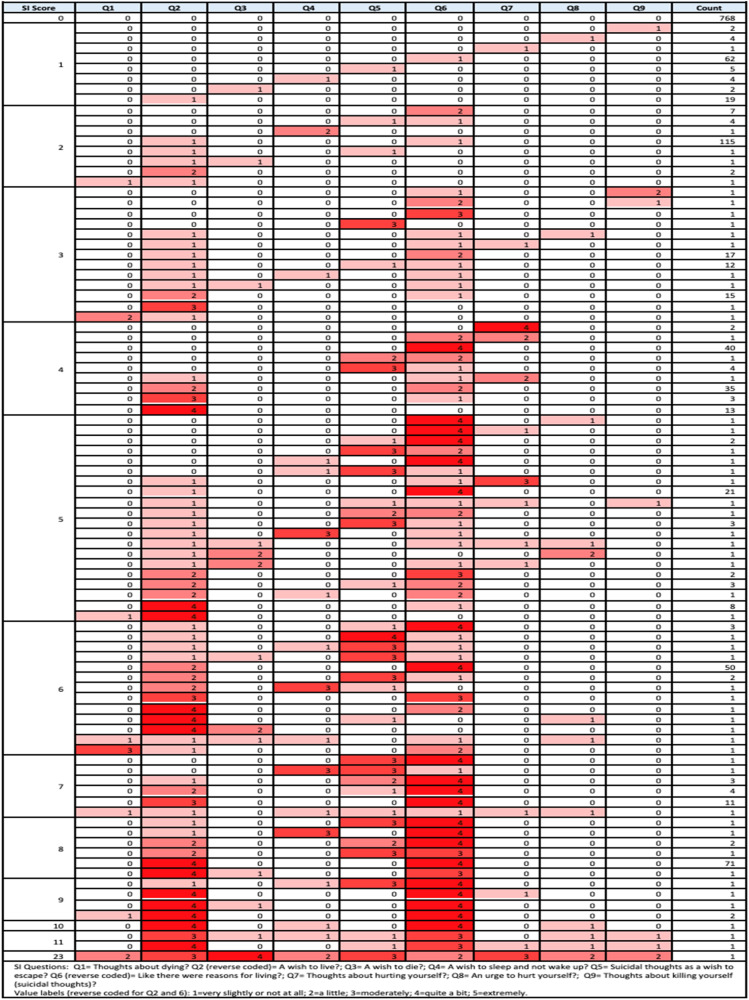
Heat map depicting relationship between suicidal ideation total scores and individual items driving the score for healthy volunteers.

HVs most often endorsed decreased reasons for living, which occurred in 544 (40%) epochs, and decreased wish to live, which occurred in 457 (33%). Endorsements of these items were unrelated to the occurrence of stressors. The next most commonly endorsed item was “suicidal thoughts as a wish to escape” reported in 5% of epochs (*n* = 68), followed by “a wish to sleep and not wake up’ (*n* = 20; 1.5%). Endorsement of other items was more sporadic and occurred in less than 1% of epochs. Amongst actual SI items, HVs reported 9 epochs with “thoughts of dying,” 14 epochs with “a wish to die,” 20 epochs with “a wish to sleep and not wake up,” 68 epochs with “suicidal thoughts as a wish to escape,” 15 epochs with “thoughts about hurting yourself,” 15 epochs with “an urge to hurt yourself,” and 8 epochs with “thoughts about killing yourself.” Thus, HVs endorsed no SI in 768 epochs (56%); only SI-related items in 488 epochs (35%), and actual SI in 158 epochs (11.5%). See Fig. 1 for a heat map depicting the distribution of SI item scores across total scores.

### Characteristics of suicidal ideation in MDD patients

Patients most often endorsed SI-related items such as decreased reasons for living (*n* = 1741 epochs 76%) and decreased wish to live (*n* = 1678; 73%). Actual SI items were endorsed less often: “suicidal thoughts as a wish to escape” (*n* = 1402; 61%); “a wish to sleep and not wake up” (*n* = 724; 32%); “a wish to die” (*n* = 445, 19%); “thoughts of dying” (*n* = 421 18%); “thoughts about hurting yourself” (*n* = 276, 12%); and “thoughts about killing yourself” (*n* = 249; 11%); and “an urge to hurt yourself” (*n* = 228, 10%). Thoughts of killing oneself and thoughts of hurting oneself or urges to do so only became salient once the total SI score was greater than 18 (data not shown).

### Impact of stressors on SI in HVs and patients

Longitudinal mixed effects logistic regression models indicated that there were greater odds that a patient would report a stressor relative to HVs for 7 of the 8 event types assessed; odds ratios ranged from 3.5 (CI = 2.4–5.0) to 17.5 (CI = 3.1–37.8) (*P* < 0.001 for all but one). This was not the case for “compliments,” which were reported by HVs and patients for 22.2% and 23.5% of epochs, respectively (see Table 2).

Mixed effects linear regression models indicated that HVs showed minor total SI increases only in the context of “neglect” (uncorrected for multiple comparisons), while patients showed increases in total SI in response to all but one stressor (“compliments”), and 6 of the remaining 7 stressors had a significant impact on SI scores (“loss” was at a trend level). However, for most events, the difference between patients and HVs in terms of the effect of stressors on total SI scores during a given epoch was not significant, except that “disagreements” had significantly more impact on patients’ SI than on HVs’ SI (See Table 3).

**Table 3:** Effects of stressors on suicidal ideation.

		Patients with MDD			Healthy volunteers			Difference				
#	Event	Est	SE	*p*	Est	SE	*p*	Est	SE	DF	*t*	*p*
120	Disagreement	1.04	0.25	<.0001	0.34	0.24	0.1562	0.7	0.35	2740	2.01	0.0442
120	Rejection	0.81	0.28	0.0043	0.33	0.57	0.5642	0.48	0.64	2740	0.75	0.4506
120	Interpersonal disappointment	0.65	0.18	0.0003	0.49	0.28	0.0799	0.16	0.33	2740	0.49	0.6222
120	Compliment	−0.27	0.17	0.1084	−0.01	0.09	0.9123	−0.26	0.19	2740	−1.34	0.1789
120	Neglect	0.73	0.19	<.0001	0.79	0.32	0.0147	−0.06	0.37	2740	−0.16	0.8759
120	Loss	0.45	0.27	0.0979	0.49	0.36	0.1782	−0.04	0.46	2740	−0.08	0.9323
120	Bad news	0.74	0.24	0.0021	0.44	0.3	0.1507	0.31	0.39	2740	0.79	0.4282
120	Painful reminder	0.29	0.15	0.0472	0.22	0.23	0.3407	0.07	0.28	2740	0.26	0.7973

# number of observations, *Est* estimate, *SE* standard error, *DF* degrees of freedom, *MDD* Major Depressive Disorder.

## Discussion

Our findings that HVs endorsed SI-related items in 44% of epochs, and actual SI items in 11.5% of epochs, or 25% of epochs with non-zero scores, suggests a much higher prevalence of SI in HVs than previously recognized. The thoroughness of the diagnostic assessment by highly experienced clinicians argues against a contribution of unrecognized psychiatric illness in this sample. That HVs sometimes endorse SI and SI-related items has not been a focus in the literature. While the frequency of SI in general populations has been reported [3, 4], to our knowledge, there are no epidemiologic reports on SI prevalence in those who do not meet criteria for a psychiatric disorder. Moreover, studies that document SI in HV often do not comment upon this finding. For example, studies including HVs with no psychiatric diagnoses occasionally document that HVs had current [11, 14–18, 32] or lifetime SI scores [12, 14, 32] greater than zero or reported past suicidal behavior [33], but do not address it in the discussion. Further, many studies do not report on SI scores in HVs [34–37], referring to variables about SI and suicidal behavior as “not applicable” or simply leaving out the information from tables and text. This may reflect a common assumption that those who do not meet criteria for psychiatric illness do not experience SI or engage in suicidal behaviors.

A number of studies, including our previous publications, report SI scores of zero in HVs [9, 10, 38, 39]. Our current findings using fine-grained data acquired through real-time EMA measures suggest a hitherto unsuspected level of SI among healthy individuals that may not have been detected if ascertained retrospectively. That said, the frequency and intensity of SI was significantly less than that of patients, suggesting that HVs are not as vulnerable to suicidal thinking, perhaps a mark of resilience. This study extends the literature by describing not only the occurrence, but the characteristics of SI in HVs, as well as its relationship to stress.

It is notable that the most frequently endorsed SI items have the same rank order in the MDD and HV group, with waning reasons for living being the most frequent in both. Cross-sectional as well as longitudinal studies using EMA report that reasons for living (RFLs) are protective against SI within subjects [40, 41], such that as RFLs decrease, SI increases, although not all studies agree [42, 43]. Given their putative protective role, it is not altogether surprising that RFLs are the first to erode, waning even at low SI total scores. The next most common items in both groups are decreased wish to live, suicidal thoughts as a wish to escape, and a wish to sleep and not wake up. These data suggest that for both HVs and patients, SI often manifests as what could be construed as passive SI (decreased wish to live). This may be viewed as clinically reassuring and, indeed, patients with “only” passive SI are generally not considered at high risk for suicide. However, a national epidemiologic study [44] suggests that the presence of “desire for death,” another type of passive SI, is associated with risk of suicide attempts, even when suicidal ideation per se (i.e., thinking about killing themselves) is absent, and some reports indicate that desire for death may predict suicide death [45, 46]. Similarly, a prospective study showed that the ratio of wish to die: wish to live predicted suicide, but not death from natural causes, underscoring the impact of low wish to live on suicide risk [47].

Stressors appear to be more common among MDD patients than HVs, which might be expected, especially if they are a consequence of psychiatric symptoms [48]. However, MDD patients are reported to be more sensitive to the environment [49] and thus may be more likely than HVs to construe events that others may view as neutral as “disappointments” or “neglect.” They would certainly be more vulnerable to “painful reminders from the past” as such ruminative thinking is commonly observed in MDD. In addition to reporting more stressors, patients with MDD demonstrated significantly greater SI in response to most stressors we measured, as we and others have reported [48, 50, 51]. In contrast, HVs reported fewer stressors than patients and were resilient to them, in that their increases in SI in response to them were minimal, except for “losses,” after which HVs tended to show small increases in SI.

HVs sometimes endorsed not having robust or sometimes any, reasons for living or that they did not have much of a wish to live, unrelated to stressors other than neglect. It may be that HVs experienced stressors not captured by the study’s prompts or that these thoughts occurred spontaneously, possibilities that our data does not address. Qualitative research may assist in clarifying this question and provide further insight into how and why HVs experience SI.

The stressors assessed in this study are not particularly severe and could be viewed as quotidian. Moreover, they are largely interpersonal in nature. A key question is whether more severe stressors would elicit higher SI scores in HVs. We do not have data to address this possibility, but US national data find that, among males, 60% of suicides occur in those with no known mental condition [52], and in such cases, stressors prior to suicide are observed more often than for decedents with known mental conditions. We posit that the presence of low levels of SI amongst HVs may augur suicide attempts or suicide in the context of overwhelming stress.

Our findings uncover a thorny problem. How are we to identify persons at risk for suicidal behavior who do not have psychiatric diagnoses? While the answer is unknown, one possible strategy may arise from studying SI using dimensional approaches. For example, it is possible that SI emerges in individuals with low distress tolerance (DT) and high intolerance of uncertainty (IU) which contribute to emotion dysregulation and/or in those with greater hypothalamic-pituitary-adrenal axis reactivity. Measures of such constructs may permit more accurate identification of risk independent of the presence of psychopathology. Indeed, these strategies are exactly the ones our team has used to study suicidal behavior with a focus on biological and cognitive features of those at risk

### Limitations

Our study examined a sample of convenience and therefore is likely not representative of the population of MDD. Moreover, since HVs were carefully screened to exclude psychiatric diagnoses including addictions, the HV group is clearly not representative of the general population. Because our sample is not representative, bias impacting the manifestation of SI in HVs is possible. For example, these HVs may be outliers in the frequency of their SI, may have signed up for studies of suicidal behavior because they had SI, or may have been particularly open to reporting SI. It also is possible that dimensional approaches to phenomenology might uncover biological or cognitive factors that underpin the occurrence of SI irrespective of the presence of a diagnosis and the lack of biomarker data in this study is a limitation. Given that EMA was, on average, only collected once within a 2-h epoch, the data is not granular enough to examine within-subject trajectories of SI. Such analyses might determine whether SI differs between patients and HVs and if so, whether the presence of MDD leads to more “active SI” as SI scores increase, for example. As well, the burdensome nature of EMA may have impacted participant engagement and led to the wide distribution of EMA responses, a limitation exacerbated by the study’s relatively small sample size. Of note, the sample is considerable given this is an EMA study. Also, HV and patient samples both had a preponderance of females who are more likely to report SI and therefore it is possible that the SI patterns described are characteristic of females and not males. Moreover, not all diagnoses are covered in the SCID I (ADHD or pathological gambling) and a study of suicides with no Axis I diagnosis [53] noted that many of those decedents may have had pathological gambling or personality disorders. Finally, because stressors were only monitored over a 7-day period, we may not have captured events severe enough to elicit higher SI scores in HVs.

### Conclusions

HVs had non-zero scores on SI-related items in 44% of epochs and endorsed actual SI items in 25% of epochs with non-zero scores. For patients, only 14% of epochs had no SI. The most frequently endorsed items were decreased reasons for living, decreased wish to live, suicidal thoughts as a way to escape, and wishing to sleep and not wake up, in that order, for both HVs and patients. HVs were less likely than patients to report or be affected by stressors and showed minor SI increases only when reporting experiencing neglect. In contrast, patients exhibited SI increases in response to 7 of the 8 stressors. That SI is observed in HVs carefully screened for Axis I and II disorders suggests that SI occurs in psychiatrically healthy individuals, albeit in a less frequent and intense manner than among patients. This implies that screening for suicide risk that is focused on those with mental disorders may be too narrow an approach.

## Data Availability

Data will be made available upon reasonable request.
